# Socioeconomic Determinants of Melanoma-Related Health Literacy and Attitudes Among College Students in China: A Population-Based Cross-Sectional Study

**DOI:** 10.3389/fpubh.2021.743368

**Published:** 2021-11-11

**Authors:** Tianhao Wu, Xianggui Wang, Shuang Zhao, Yi Xiao, Minxue Shen, Xi Han, Xiang Chen, Juan Su

**Affiliations:** ^1^Department of Dermatology, Xiangya Hospital, Central South University, Changsha, China; ^2^Hunan Engineering Research Center of Skin Health and Disease, Central South University, Changsha, China; ^3^Hunan Key Laboratory of Skin Cancer and Psoriasis, Central South University, Changsha, China; ^4^Eye Center of Xiangya Hospital, Central South University, Changsha, China; ^5^ULink College Guangzhou, Guangzhou, China

**Keywords:** melanoma, health literacy, mediation model, attitude, prevention

## Abstract

**Objectives:** To investigate the association of gender, ethnicity, living region, and socioeconomic status (SES) with health literacy and attitudes toward nevi and melanoma in Chinese adolescents and to examine whether health literacy mediates the association of SES with attitudes.

**Study Design:** A multicenter cross-sectional study was conducted among newly enrolled college students. First-year students were recruited from five universities in different regions of China in 2018 using the cluster sampling method. The observers were blinded to the participants.

**Methods:** Health literacy and attitudes were measured using a previously validated tool (*Nevus and Melanoma Health Literacy and attitudes Test*). SES was measured by annual family income and parental highest educational level. Nonparametric test was used to examine the association of participants' characteristics with health literacy and attitudes. Two-level generalized linear model with logarithm link function and Gamma distribution was used individually for SES. The mediation effect model was used to examine the mediation effect of health literacy.

**Results:** A total of 21,086 questionnaires were completed by college students with a mean age of 18.0 ± 0.8 years. The mean scores of health literacy and attitudes were 9.83 ± 7.46 (maximum score: 28) and 16.98 ± 2.92 (maximum score: 20), respectively. Female, Han nationality, annual family income, and parental educational levels were positively associated with health literacy and attitudes. Regional differences showed different effects on health literacy and attitudes. A mediation model showed that literacy mediated the association of SES with attitudes toward nevi and melanoma. Health literacy mediated ~30–50% of the association of SES with attitudes.

**Conclusions:** Melanoma-related health literacy among Chinese college students is generally insufficient and needs to be improved. Targeted and personalized health education for improving health literacy related to nevi and melanoma may improve the general population's attitudes and further promote health-related behavior to prevent and identify early-stage melanoma.

## Introduction

Melanoma is the most serious form of skin cancer. It is characterized by easy metastasis, difficult treatment, and poor prognosis. Additionally, the economic burdens for late-stage melanoma associated with treatments, adverse treatment events, death, and disability are also heavier than other skin cancer, like squamous cell carcinoma and basal cell carcinoma (1–3). According to the Surveillance, Epidemiology, and End Results (SEER) database, from 1992 to 2018, the number of new cases was increasing and the death rates were falling in the US (4). The morbidity and mortality rates of melanoma in China were 0.48 and 0.20/100,000, respectively, in 2011 (5). Prognosis for people with melanoma depends on the stage of the disease at the time of diagnosis. Most people with stage I melanoma can expect prolonged disease-free survival and can likely be cured, whereas those with more advanced stage II–IV disease are more likely to develop metastatic disease (6, 7).

Considering the high fatality and financial burden, it is necessary to propose a strategy to improve the early diagnosis rate and prognosis of melanoma. It has been reported that primary and secondary prevention strategies for melanoma, such as clinician screening, self-examination, and sun protection, can significantly reduce the risk of advanced melanoma and fatality (8–11). It is clear that melanoma in Caucasians is associated with sun exposure, and sun protection can significantly reduce the risk of melanoma. However, sun protection may be largely ineffective for the Chinese population because the most common type of melanoma in China is acral lentiginous melanoma, which is not related to sun exposure. But another way to prevent may be useful. A case-control study of the U.S. showed that individuals reporting skin awareness (thinking about changing of one's skin appearance before go to a doctor) had a lower risk of death from melanoma than those without reporting skin awareness (12). The study also found that 15% of melanoma patients practicing self-examination had a lower risk of advanced melanoma (10, 12). Another study involving 566 newly diagnosed melanoma patients suggested that routine skin self-examination of some or all of the body was associated with nearly twice the possibility of a thin ( ≤ 1 mm) melanoma at diagnosis compared with no self-examination (13). Thus, the benefits of self-examination can be clearly seen.

Socioeconomic status (SES), an important indicator of socioeconomic determinants, in studying health disparities across different populations is often measured according to income, education level, and occupation at the individual level and is determined by diverse economic contexts at the population level (14–16). A study reported that people with higher incomes and higher levels of education have a higher risk of developing melanoma, and they are less likely to be diagnosed with later-stage melanoma compared with their lower SES counterparts (17). In a recent study involving 261,076 participants, SES was divided into three groups, and low SES was found to be associated with low survival. The melanoma-specific survival of the low SES group is significantly worse than that of the high SES group (18). At the same time, a published review reported that one of the potential mediating factors that link SES and health is health literacy (19). Health literacy was defined by World Health Organization as cognitive and social skills that determine individuals' motivations and abilities to acquire, understand, and use information, and to promote and maintain health. It refers to the ability to acquire and understand information. It is obviously related to knowledge. Although SES can not easily be changed, health education and promotion targeting health literacy may provide an opportunity to change health-related attitudes and behavior. Therefore, some personal or family factors, including SES, may be predictors to identify students with low health literacy and negative attitudes. The improvement of health literacy may be one of the ways to improve students' melanoma-related attitudes.

In summary, to understand the residents' health literacy and attitudes toward melanoma, we conducted a survey on Chinese college students about their health literacy of discovering early melanoma and proper handling. By analyzing the relationship between students' characteristics including SES, health literacy, and melanoma-related attitudes, we attempt to find a way to encourage residents to actively and correctly carry out self-examination. We also hope to provide evidence-based support for the government and health departments to formulate health promotion public health policies.

## Methods

### Study Design and Participants

**Figure 1:**
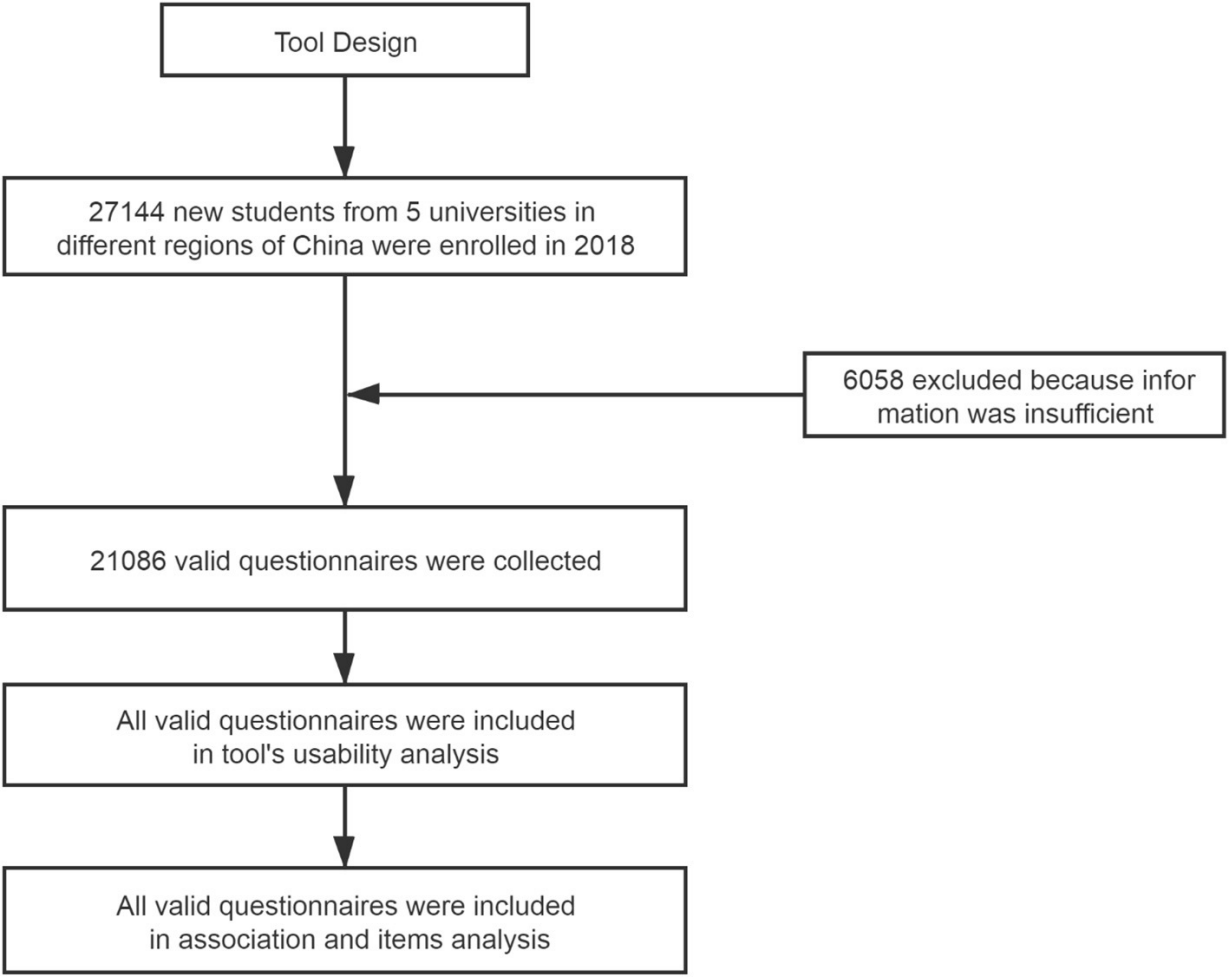
Investigation profile.

This was a cross-sectional study. Five universities in different regions of China were selected. Twenty-seven thousand and one hundred and forty-four first-year college students who consented to participate completed an online questionnaire survey after their enrollment at the universities in 2018. The questionnaire survey was administered with the supervision and coordination of the departments of student affairs of the universities. More details of about the College Student Skin Health Study (CSSHS) can be found in the pilot study conducted in 2017 (20). The investigation profile is shown in Figure 1.

Melanoma-related health literacy and attitudes were measured by a previously validated tool (Supplementary Material 1) (21). The validity reliability had been already made. The tool includes two subscales to measure literacy (A1–C3) and attitudes (D1–D5). Demographic information, including age, gender, ethnicity, annual family income, and highest parental educational level, was also collected through the questionnaire survey. The maximum scores of the health literacy subscale and attitudes subscale were 23 and 20, respectively. If the score reaches more than 80% of the total score, it will be regarded as qualified.

SES was measured by annual family income and highest parental educational level. Because all of the participants were newly enrolled students, we used parental SES to represent the socioeconomic strata of their original families. Annual family income was categorized as below US$1,450; 1,450–4,347; 4,348–7,246; 7,247–14,492; 14,493–28,985, and above 28,985 (exchange rate in 2018: 1 USD = 6.9 CNY). Highest parental educational level was categorized into primary school and below, middle school, high school, college, and above.

This study was conducted according to the guidelines of the Declaration of Helsinki. All procedures involving participant were approved by the institutional research ethics board of Xiangya Hospital, Central South University (Changsha, China). Informed consent was obtained from all participants.

### Statistical Analysis

Due to the uneven variance, the sample size needed to compare the health literacy (attitudes) of different demographic factors was calculated according to the sample size estimation method of Satterthwaite *t*-test. The mean and standard deviation of the health literacy subscale (A1–C3) and attitudes subscale (D1–D5) scores were presented and compared using the Mann Whitney *U*-test and Kruskal–Wallis test. Owing to the variance structure related to cluster sampling, two-level generalized linear models with a Gamma distribution and a logarithm link function were used to fit the data, where individuals (level 1) were nested within universities (level 2). The intracluster correlation coefficient (ICC) and model fit (measured by the Akaike information criterion, AIC; and Schwarz's Bayesian information criterion, BIC) were examined. The models were adjusted for the fixed effects of demographic variables, including age and gender, and the random effects of the university. B1 (early identification of melanoma) and C3 (a behavioral risk factor for melanoma) were selected for item analysis using the same method. Mediation analysis was performed to investigate whether SES affects attitudes through literacy. The model with a mediation effect was proposed as SES (X1: income; X2: education) → literacy (M) → attitudes (Y). Statistical analysis was performed in SAS 9.4 (SAS Inc., Cary, USA). *P* <0.005 was considered statistically significant for multiple comparisons.

## Results

A total of 21,086 valid questionnaires were completed by 27,144 newly enrolled students (response rate 78%). The mean age was 18.0 ± 0.8 years (female: 49.3%, male: 50.7%). The distribution of ethnicity, ethnicity, region, and SES indicators are shown in Table 1.

**Table 1:** Distribution of participant characteristics.

**Characteristics**	***N* (%)**	**Literacy (Mean ± SD)**	**Attitudes (Mean ± SD)**
**Gender**			
Male	10,700 (50.7)	9.32 ± 7.675	16.66 ± 3.127
Female	10,386 (49.3)	10.84 ± 7.432	17.44 ± 2.629
**Ethnicity**			
Han	16,881 (80.1)	10.48 ± 7.699	17.09 ± 2.895
Other	4,205 (19.9)	8.42 ± 6.919	16.88 ± 3.006
**Region**			
North	3,779 (17.9)	9.86 ± 7.423	17.11 ± 2.900
Northeast	651 (3.1)	10.04 ± 7.821	16.95 ± 3.365
East	4,590 (21.8)	10.88 ± 7.985	17.22 ± 2.970
Central	4,368 (20.7)	10.27 ± 7.764	16.90 ± 2.871
South	1,476 (7.0)	10.72 ± 7.732	16.80 ± 2.918
Southwest	1,876 (8.9)	10.43 ± 7.518	17.01 ± 2.759
Northwest	4,346 (20.6)	8.81 ± 6.902	17.08 ± 2.912
**Family annual income (USD)**			
<1,450	2,317 (11.0)	8.63 ± 7.162	16.72 ± 3.040
1,450–4,347	4,630 (22.0)	9.39 ± 7.225	16.85 ± 2.916
4,348–7,246	3,633 (17.2)	9.98 ± 7.450	17.02 ± 2.801
7,247–14,492	4,568 (21.7)	10.61 ± 7.699	17.15 ± 2.870
14,493–28,985	4,229 (20.0)	10.85 ± 7.825	17.26 ± 2.929
>28,985	1,709 (8.1)	10.7 ± 8.125	17.28 ± 3.035
**Parental highest education**			
Primary school and below	1,405 (11.0)	8.59 ± 7.084	16.56 ± 3.033
Middle school	5,584 (26.5)	9.46 ± 7.207	16.80 ± 2.914
High school	5,293 (25.1)	9.91 ± 7.482	17.08 ± 2.883
College and above	8,511 (40.4)	10.86 ± 7.912	17.28 ± 2.898
Unknown	293 (1.4)	8.73 ± 7.255	16.64 ± 3.002

### Association of Participant Characteristics With Literacy and Attitudes

**Figure 2:**
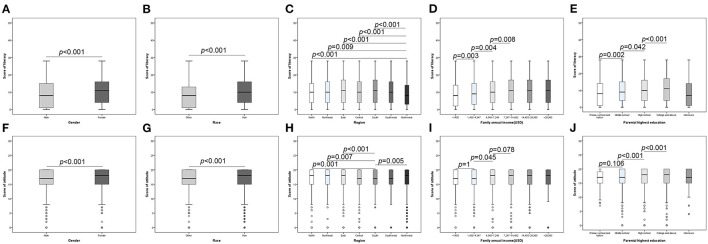
Association between literacy (attitudes) and participant characteristics. **(A)** Health literacy between different gender; **(B)** Health literacy between different races; **(C)** Health literacy between different regions; **(D)** Health literacy between different family annual income; **(E)** Health literacy between different races; **(F)** Attitudes between different gender; **(G)** Attitudes between different races; **(H)** Attitudes between different regions; **(I)** Attitudes between different family annual income; **(J)** Attitudes between different races.

Female and Han participants obtained higher literacy and attitudes scores compared with men and other ethnicity (Figures 2A,B,F,G). The mean literacy score of students from the northwest was 8.81 ± 6.902, which was significant lower than others. The difference of attitudes scores in different regions is relatively less significant. Only students from the south seems to have a more negative attitudes (Figures 2C,H). Family income and parental education showed a similar effect to literacy and attitudes, that with the increase of family income and parents' education level, the literacy and attitudes score gradually increased (Figures 2D,E,I,J).

### Association of SES With Literacy and Attitudes

The mean health literacy score was 9.83 (35.1% of the total score 28), and the mean attitudes score was 16.98 (84.9% of the total score 20). The ICCs of literacy and attitudes were very low at the university level (0.51–1.09%), indicating minimal center effects (Table 2). The generalized linear models showed a better model fit (smaller AIC, BIC, and χ^2^/df) than linear models (data not shown). According to the standardized coefficients, which are shown in Table 2, parental education (βincreased from 2.81 to 9.45 for literacy and from 0.69 to 2.44 for attitudes) showed a stronger effect than family income (βincreased from 2.09 to 5.46 for literacy and from 0.35 to 1.17 for attitudes).

**Table 2:** Associations of parental socioeconomic status with melanoma-related literacy and attitudes estimated using two-level log-Gamma models.

**Socioeconomic indicators**	**Literacy**			**Attitudes**		
	**Mean (SD)**	**β (SE)^**a**^**	***P***	**Mean (SD)**	**β (SE)^**a**^**	***P***
**Ethnicity**						
Han	10.29 (7.58)	Reference		17.02 (2.89)	Reference	
Other	8.19 (6.80)	−8.79 (0.78)	<0.001	16.85 (3.01)	−1.04 (0.19)	<0.001
**Family annual income (USD)**						
<1450	8.68 (7.17)	Reference		16.73 (3.03)	Reference	
1,450–4,347	9.37 (7.23)	2.09 (1.06)	0.049	16.85 (2.92)	0.35 (0.25)	0.157
4,348–7,246	10.02 (7.45)	3.72 (1.03)	<0.001	17.03 (2.80)	0.71 (0.24)	0.003
7,247–14,492	10.62 (7.68)	5.46 (1.12)	<0.001	17.17 (2.86)	1.04 (0.26)	<0.001
14,493–28,985	10.36 (7.55)	4.83 (1.14)	<0.001	17.09 (3.06)	1.17 (0.27)	<0.001
>28,985	9.86 (7.92)	2.08 (0.94)	0.028	17.01 (3.12)	0.84 (0.22)	<0.001
**Parental highest education**						
Primary school and below	8.59 (7.08)	Reference		16.56 (3.03)	Reference	
Middle school	9.46 (7.21)	2.81 (1.32)	0.033	16.80 (2.91)	0.69 (0.31)	0.026
High school	9.91 (7.48)	4.50 (1.33)	<0.001	17.08 (2.88)	1.66 (0.31)	<0.001
College and above	10.67 (7.80)	9.45 (1.51)	<0.001	17.23 (2.89)	2.44 (0.35)	<0.001
**Model evaluation**						
ICC (%)		1.09			0.51	
χ^2^/df		0.47			0.03	
AIC		43,863			17,213	
BIC		43,862			17,210	

*SE, standard error; ICC, intracluster correlation coefficient; AIC, Akaike information criterion; BIC, Schwarz's Bayesian information criterion.*

a *Standardized regression coefficients, adjusted for the fixed effects of age, gender, and region, as well as the random effect of university*.

### Medication Effect

**Figure 3:**
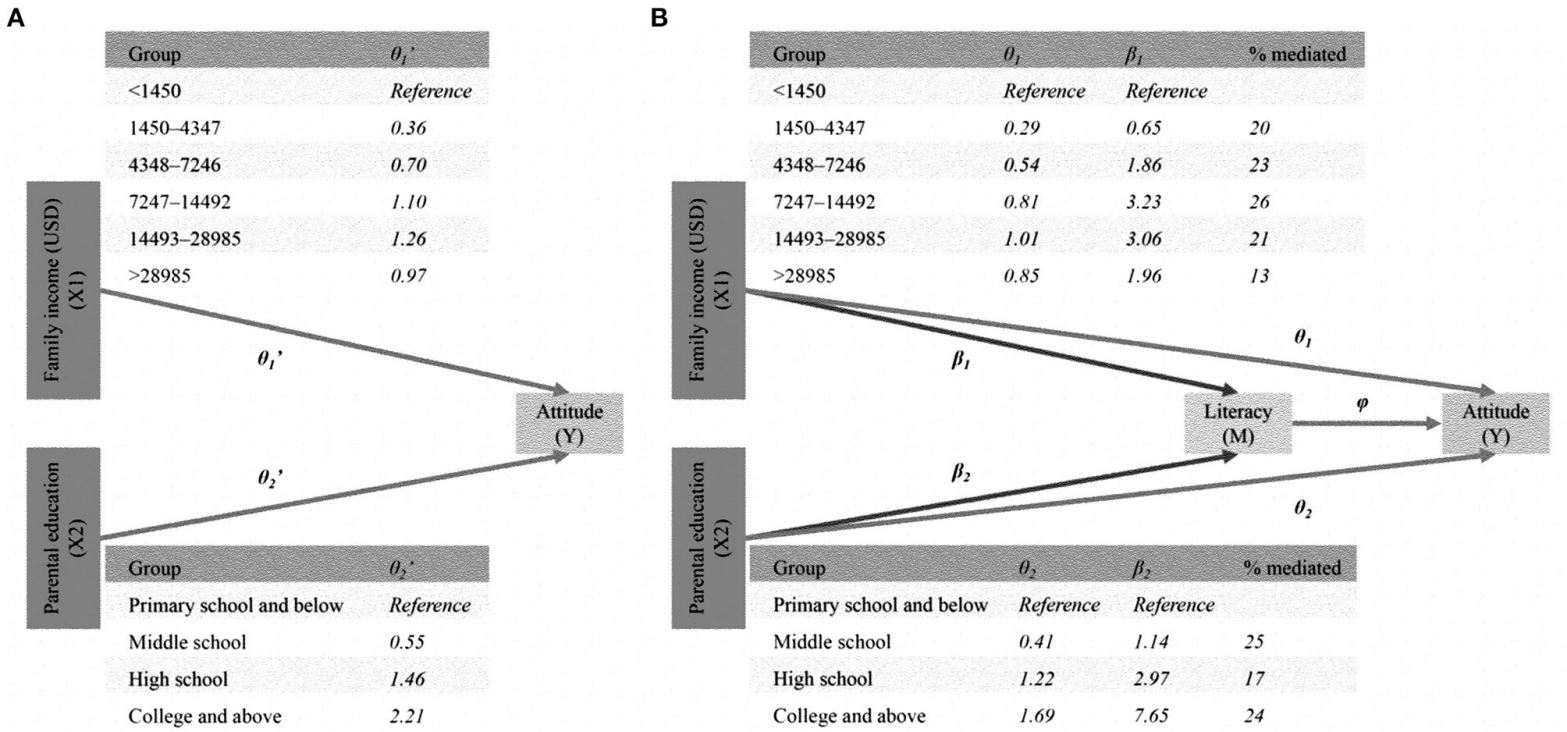
Medication model: SES (X1: income; X2: education) → literacy (M) → attitudes (Y). **(A)** Total effect. **(B)** Direct and medication effect.

The direct effects (θ1 and θ2 in Figure 3B) of SES were significantly smaller than the total effects (θ1′ and θ2′ in Figure 3A). Literacy mediated 13–26% of the effect of income and 17–25% of the effect of parental education. Taken together, literacy mediated ~30–50% of the total effect of SES on attitudes.

### Item Analysis

B1 (the “ABCDE” criteria for early identification of melanoma) and C3 (a behavioral risk factor for malignant transformation of nevi) were the key items of health literacy and were selected for item analysis. The average scores of items B1 and C3 were both <2. The correct rates are lower (B1 compared with B2 and C3 compared with C1 and C2). Consistent with the above result, SES was positively correlated with both B1 and C3 scores (Table 3). Parental education (β increased from 2.52 to 6.73 for literacy and from 2.53 to 7.63 for attitudes) also showed a stronger effect size than family income (β increased from 3.68 to 5.95 for literacy and from 1.54 to 4.34 for attitudes).

**Table 3:** Item analysis.

**Socioeconomic indicators**	**Item B1 (range: 0–4)**			**Item C3 (range: 0–4)**		
	**Mean (SD)**	**β (SE)^**a**^**	***P***	**Mean (SD)**	**β (SE)^**a**^**	***P***
**Ethnicity**						
Han	1.56 (1.70)	Reference		1.36 (1.37)	Reference	
Other	1.04 (1.40)	−8.34 (0.79)	<0.001	1.01 (1.51)	−5.51 (0.68)	<0.001
**Family annual income (USD)**						
<1,450	1.14 (1.49)	Reference		1.06 (1.20)	Reference	
1,450–4,347	1.38 (1.62)	3.68 (1.02)	<0.001	1.19 (1.29)	1.54 (0.89)	0.084
4,348–7,246	1.52 (1.67)	5.41 (0.99)	<0.001	1.35 (1.35)	4.34 (0.87)	<0.001
7,247–14,492	1.59 (1.71)	5.95 (1.08)	<0.001	1.39 (1.39)	4.07 (0.94)	<0.001
14,493–28,985	1.62 (1.73)	4.92 (1.11)	<0.001	1.45 (1.42)	4.18 (0.96)	<0.001
>28,985	1.53 (1.74)	2.35 (0.92)	0.142	1.43 (1.46)	1.89 (0.80)	0.018
**Parental highest education**						
Primary school and below	1.20 (1.53)	Reference		1.07 (1.24)	Reference	
Middle school	1.40 (1.62)	2.52 (1.28)	0.048	1.23 (1.30)	2.53 (1.11)	0.023
High school	1.47 (1.67)	3.32 (1.28)	0.01	1.29 (1.33)	3.33 (1.12)	0.003
College and above	1.60 (1.73)	6.73 (1.46)	<0.001	1.45 (1.43)	7.63 (1.27)	<0.001

*SE, standard error.*

a *Standardzed regression coefficients, adjusted for the fixed effects of age, gender, and region, as well as the random effect of university*.

## Discussion

In our study, limited health literacy but a qualified attitudes toward melanoma were observed among first-year college students in China. We found that SES was positively correlated with the level of literacy and attitudes toward melanoma. In detail, Female, Han nationality, annual family income and parental educational levels were positively associated with health literacy and attitudes. Regional differences showed different effects on health literacy and attitudes. The health literacy of students in Northwest China is relatively poor compared with other regions. The melanoma-related attitudes of students in the south is more negative than that in other regions. By mediation effect analysis, we then find that melanoma-related health literacy mediated the effect of SES on attitudes. According to these results, we raised a point that gender, ethnicity, region, annual family income, and Parental highest education can be predictors of Chinese students' health literacy and melanoma-related attitudes, and health literacy enhancement can promote a more positive attitudes. Interestingly, we found that the melanoma-related health literacy of Chinese adolescents was generally insufficient, but the attitudes toward prevention and health education were better. One reason for this phenomenon may be that although the population has limited knowledge about melanoma, skin diseases that cause pigmentation, ulceration or scarring may attract people's attention owing to the issue of disfigurement. Another explanation may be that the content of the questionnaire contains information about melanoma, which may have some effects on the participants. In addition, we observed a lower accuracy level of item B1 (compared to B2) and C3 (compared to C1 and C2) than other items of melanoma-related health literacy. Item B1 requires knowledge about the ABCDE criteria for the early identification of melanoma, and for item C3, we asked about the behavioral risk factors for malignant transformation of nevi. These two aspects should be a focus when implementing health education.

First, there may be delayed diagnosis among Chinese patients with melanoma compared with the data from SEER (5). Certainly, early diagnosis can reduce the fatality of melanoma. It has been reported that the reasons for delays in melanoma diagnosis are multifactorial, and the patient factor (e.g., a lack of knowledge and a failure of patients to examine the skin routinely) seems to be the most important among previous studies (22–25). In a study analyzing the diagnostic delay in 211 patients with cutaneous melanoma, the main factor of delay was related to patients (26). It also reported that only 31.3% of the patients knew that melanoma was serious skin cancer, and most people had insufficient knowledge about melanoma, which led to delayed diagnosis and treatment (26). We also observed a lack of melanoma-related health literacy among Chinese adolescents. Although the population is limited to college students and a broader investigation is required, it is reasonable to speculate that the deficiency may impact the opportunity for early identification of melanoma. The preceding study also found that inappropriate previous treatment (e.g., ointment, electrocautery, liquid nitrogen, and curettage) without histopathological confirmation occurred in 14.7% (31/211) of the involved patients (26). What we also find is ignorance among the participating population when asking item C3 (Please choose which of the following incentives can facilitate the malignant transformation from nevus to melanoma), which is about the behavioral risk factor for malignant transformation of nevi and proper treatment of melanoma.

SES is a well-established social determinant of health and disease. Higher SES is related to a better prognosis of melanoma (17, 18). A study of non-Hispanic whites was conducted using SEER data from 1992 to 2004 and showed that individuals with low SES exhibited a higher increase in melanoma incidence than individuals with a higher SES (27). The current study proved that melanoma-related health-literacy mediated the effect of SES on attitudes in Chinese college students. The association of SES with health literacy was consistent with previous studies. A cross-sectional study in South China found that educational level was a significant social determinant for general health literacy, and its effect size was much stronger than that of occupation and income (28). Educational level, income, and physical limitations were identified as factors with the strongest contribution to inadequate or problematic health literacy in the Catalan population (28). A multicenter study in European countries showed that subgroups within their study population, defined by financial deprivation, low social status, low education, or old age, had higher proportions of people with limited health literacy (29). The authors concluded that limited health literacy represented an important challenge for health policies and practices across Europe but to a different degree for different countries. Although SES is not a modifiable risk factor in the short term, the social gradient in health literacy should be taken into account when developing public health strategies to improve health equity.

Compared with SES, health literacy could be improved through health education and promotion. According to the mediation effect model, we identified 30–50% effect mediation of the association of SES with attitudes by health literacy, indicating that health literacy might serve as a modifiable factor that intermediates health-related attitudes and even behavior that directly affect health outcomes. Previous studies have suggested that primary and secondary prevention strategies are effective ways to reduce the mortality of melanoma (8–11, 30, 31) attitudes toward melanoma are entwined with early prevention behavior (32). In this regard, an intervention targeting the risk factors and early recognition of melanoma through health education might be a cost-effective approach to improving health literacy and health outcomes of melanoma, especially in male, non-Han, south, and northwest students with low SES.

Many governments around the world have taken some public health measures to reduce the risk of skin cancer, including melanoma. For example, Australia's SunSmart campaign and the U.S. Environmental Protection Agency's SUNWISE program (33). Although no public health plan has been linked to the incidence rate of melanoma, many studies have found that health education can improve the prevention behavior of melanoma patients. At present, there is no research on melanoma-related health education in China. Thus, we hope to provide suggestions for melanoma prevention strategies in China.

Health education for different students should be adjusted accordingly. It should emphasize more on the early symptoms and signs of melanoma, such as the “ABCDE” criteria and proper treatment of early melanoma or high-risk nevi. As early as 1999, Oliveria et al. (34) investigated the relationship between knowledge, awareness, and treatment delay in 255 patients with melanoma. A total of 63.1 and 65.9% of the subjects thought that bleeding and scab were the indications of melanoma. In contrast, other early symptoms of melanoma are less likely to be associated with the discovery of melanoma by patients: color change (46.7%), increase in diameter (28.2%), and change in form (40.8%). Similar to our study, people do not correctly recognize the early symptoms and signs of melanoma in China. As mentioned above, improper handling of melanoma patients before a certain diagnosis has been reported previously (26). Combined with our clinical experience, much improper handling is carried out not only by doctors but also by patients themselves or their families. Additionally, previous studies have found that only approximately half of melanoma patients are discovered by themselves, and the other half are found by family members, friends, and doctors (35–37). It has been reported that melanoma discovered in clinical physical examination by clinicians is thinner than that found by accident by patients (38–40). This situation indicates that patients may find melanoma later and shows that knowledge about the diseases of nonprofessionals other than patients and doctors is crucial for the early diagnosis of melanoma. Therefore, although almost all students do not have high-risk nevus or melanoma, they also need better health literacy and attitudes.

To summarize, the risk factors for melanoma differ between Asians and whites. While ultraviolet radiation is a major risk factor among Caucasians, friction and extrusion might be more important risk factors among the Asian population, among whom acral melanoma is more common. We should tailor health education programs according to the ethnicity-specific characteristics of the disease in the context of China. Individualized adjustment of health education plan according to the characteristics of students. It should emphasize the presentation and proper management of early melanoma. Elevating melanoma-related health literacy level should be general.

## Conclusions

The melanoma-related health literacy among college students in China is generally limited and needs to be improved, especially the knowledge of performance and proper treatment of early melanoma. We found that health literacy mediated the association of SES with attitudes. We consider that combining with the features of melanoma among Asians, interventions tailored for ethnicity-specific nevi and melanoma-related health education may be helpful to promote the early diagnosis of skin melanoma.

## Data Availability Statement

The original contributions presented in the study are included in the article/Supplementary Material, further inquiries can be directed to the corresponding author/s.

## Author Contributions

TW and XW drafted the manuscript. TW and MS analyzed the data. TW, XW, SZ, YX, MS, XH, and JS participated in the field study. XC, MS, and JS designed the study, coordinated the field survey, and critically reviewed and revised the manuscript. All authors gave final approval to the version submitted for publication.

## Funding

This work was supported by the National Key R&D Project—Precision Medicine Program of China (2016YFC0900802), the Natural Science Foundation of China (81974478), the Department of Science and Technology of Hunan Province (2018SK2086 and 2018SK2092), and the Program of Introducing Talents of Discipline to Universities (111 Project, No. B20017).

## Conflict of Interest

The authors declare that the research was conducted in the absence of any commercial or financial relationships that could be construed as a potential conflict of interest.

## Publisher's Note

All claims expressed in this article are solely those of the authors and do not necessarily represent those of their affiliated organizations, or those of the publisher, the editors and the reviewers. Any product that may be evaluated in this article, or claim that may be made by its manufacturer, is not guaranteed or endorsed by the publisher.

## Supplementary Material

Supplementary Material 1 Study tool to measure health literacy and attitudes.
