# Supplementary material for: Socioeconomic Determinants of Melanoma-Related Health Literacy and Attitudes Among College Students in China: A Population-Based Cross-Sectional Study
Source: Front Public Health. 2021 Nov 11;9:743368. doi: 10.3389/fpubh.2021.743368 (PMC8632051; doi:10.3389/fpubh.2021.743368)
Supplement: Supplementary Material 1 — Study tool to measure health literacy and attitudes. [file Data_Sheet_1.PDF]

Original version in Chinese

中国大学生健康素养与态度测试

**情景一：**电影《非诚勿扰 2》中的场景：李香山脱下袜子，解开缠在脚上的纱布，露出一颗黑色大痣。他告诉秦奋说，这叫黑色素瘤，是自己从小就有的黑痣变的，两周前觉得不适，去医院检查才知道已变成肿瘤，而且已经恶化成绝症。
A1. 黑色素瘤是一种什么疾病（单选）？<br>○ 一种皮肤癌<br>○ 一种与皮肤无关的癌<br>○ 一种非癌性的皮肤疾病<br>○ 其他<br>○ 不清楚
A2. 出生就有的色素痣可以变成黑色素瘤吗？ *（本题经评估后被删除）*<br>○ 是<br>○ 否<br>○ 不清楚
A3. 黑色素瘤可能发生以下哪些部位（多选）？<br>□ 面部<br>□ 足底<br>□ 指（趾）甲<br>□ 外阴<br>□ 不清楚
**情景二：**请阅读下列材料后回答问题。"ABCDE"是黑色素瘤常用的判断指标，其含义如下：A-不对称性（Asymmetry）：色素痣形状大多较为对称，而黑色素瘤的形状大多不对称；B-边缘（Border）：色素痣边界多规则，清楚，而黑色素瘤的边界常不规则；C-颜色（Color）：色素痣的颜色较均一，而黑色素瘤可能会有多种颜色掺杂，深浅不一；D-直径（Diameter）：黑素瘤的直径通常大于 6 毫米；E-进展（Evolvement）：最重要的指标，指色素痣最近有无出现颜色或形状或大小的改变。如果符合"ABCDE"标准，则高度怀疑黑素瘤，需

取活检进行组织病理学检查进一步确诊。
B1. 以下哪些情况需要考虑黑素瘤的可能（多选题）？<br>□ 边界不规则<br>□ 色素痣的颜色出现黑白棕掺杂<br>□ 直径 1 月内由 1cm 增大至 2cm<br>□ 色素痣出现不对称<br>□ 不清楚
B2. 如果高度怀疑黑素瘤可能，需要做什么检查进一步确诊（单选题）？<br>○ 组织病理学检查<br>○ B 超<br>○ 抽血化验<br>○ X 片或 CT<br>○ 不清楚
**情景三：**请阅读下列材料后回答问题：黑色素瘤在白色人种和黄色人种的特征有较大差异。1、白种人中浅表扩散型黑色素瘤最多见，而黄色人种以肢端雀斑样黑素瘤多见。2、我国黑色素瘤常见类型首先为肢端型，即原发病灶位于足底、足趾、手指末端及甲下等部位；而白种人常见于背部、胸膜部和下肢皮肤。3、从病因学上看，白种人黑色素瘤的发生与长期或间歇性高强度的紫外线照射明确相关。我国黑色素瘤原发病灶多位于足跟、手掌、指趾和甲下等接触紫外线极少的地方，认为不恰当的处理如刀割、绳勒、盐腌、不断摩擦刺激、激光和冷冻等，有可能诱发色素痣的恶变和迅速生长。
C1. 我国黑素瘤好发于以下哪些部位（多选题）？<br>□ 指甲<br>□ 趾甲<br>□ 手掌<br>□ 足跟<br>□ 不清楚
C2. 你是否同意以下说法：我国黑色素瘤的发生与紫外线关系不大（单选题）<br>○ 同意

○ 不同意

○ 不清楚

C3. 下列哪些因素可以诱发色素痣变为黑色素瘤（多选）？

□ 激光点痣

□ 长期日晒

□ 长期摩擦、挤压

□ 外伤刺激

□ 不清楚

请逐条阅读以下陈述，并选择你的态度（单选题）

陈述	非常同意	同意	不确定	不同意	非常不同意
D1. 如果发现自己身上的色素痣有变化，你会考虑就医					
D2. 开展一些皮肤肿瘤的科普宣传是有必要的					
D3. 学习一些黑素瘤自我检查知识是有必要的					
D4. 如果你的不良习惯会增加黑素瘤的发生几率，你愿意从现在开始改变					
D5. 如果学校开设皮肤肿瘤知识讲座，你会积极参加					

<u>Translated version</u>

Nevus & Melanoma Health Literacy and Attitude Test

**Scenario 1 (a paragraph of information provided)**
A1. What kind of disease is melanoma?<br>○ Skin Cancer<br>○ A kind of cancer not related to skin<br>○ A non-cancer skin disease<br>○ Other<br>○ Not sure
A2. Can a congenital nevus develop melanoma? *(Removed after assessment)*<br>○ Yes<br>○ No<br>○ Not sure
A3. Melanoma can develop in which of the following locations?<br>☐ Face<br>☐ Foot<br>☐ Nail or toenail<br>☐ Vulva<br>☐ Not sure
**Scenario 2 (a paragraph of information provided)**
B1. Which of the following situations happening to a nevus should be considered malignant?<br>☐ With an irregular boundary<br>☐ A multi-colored nevus with black, brown and white<br>☐ The diameter of the nevus increases from 1cm to 2cm in a month<br>☐ The nevus becomes asymmetrical<br>☐ Not sure
B2. If melanoma is highly suspected, which of the following examination should be

done to confirm?<br>○ Histopathological examination<br>○ Ultrasonography<br>○ Blood test<br>○ X-ray or CT<br>○ Not sure
**Scenario 3 (a paragraph of information provided)**
C1. Which of the following is common site of melanoma in the Chinese population?<br>☐ Nail<br>☐ Toenail<br>☐ Palm<br>☐ Planta pedis<br>☐ Not sure
C2. Do you agree with the statement: Chinese melanoma is less relevant to ultraviolet, compared with Caucasians.<br>○ Agree<br>○ Disagree<br>○ Not sure
C3. Which of the following incentives can facilitate the malignant transformation from nevus to melanoma?<br>☐ Laser mole removal<br>☐ Long term exposure to sunlight<br>☐ Long-term squeeze and/or rub<br>☐ Injuries and ulcers<br>☐ Not sure
**Please read the following statements, and choose your attitude.**
D1. I will consider visiting a dermatologist if I find that my nevus has changed(eg. Size, color, ulceration, etc.).<br>○ Strongly agree

- Agree
- Not sure
- Disagree
- Strongly disagree

D2. Health education for skin cancer is necessary.

- Strongly agree
- Agree
- Not sure
- Disagree
- Strongly disagree

D3. It is necessary to learn more about melanoma self-examination.

- Strongly agree
- Agree
- Not sure
- Disagree
- Strongly disagree

D4. If I have some habits which may increase the risk of melanoma, I would like to change right now.

- Strongly agree
- Agree
- Not sure
- Disagree
- Strongly disagree

D5. If there are lectures about skin cancer, I would like to participate.

- Strongly agree
- Agree
- Not sure
- Disagree
- Strongly disagree
